# Footwear Decreases Gait Asymmetry during Running

**DOI:** 10.1371/journal.pone.0138631

**Published:** 2015-10-21

**Authors:** Stefan Hoerzer, Peter A. Federolf, Christian Maurer, Jennifer Baltich, Benno M. Nigg

**Affiliations:** 1 Human Performance Laboratory, Faculty of Kinesiology, University of Calgary, Calgary, Alberta, Canada; 2 Institute for Sport Science, University of Innsbruck, Innsbruck, Tyrol, Austria; 3 Department of Neuroscience, Norwegian University of Science and Technology, Trondheim, Norway; 4 Red Bull Diagnostic and Training Center, Thalgau, Salzburg, Austria; University of Zaragoza, SPAIN

## Abstract

Previous research on elderly people has suggested that footwear may improve neuromuscular control of motion. If footwear does in fact improve neuromuscular control, then such an influence might already be present in young, healthy adults. A feature that is often used to assess neuromuscular control of motion is the level of gait asymmetry. The objectives of the study were (a) to develop a comprehensive asymmetry index (CAI) that is capable of detecting gait asymmetry changes caused by external boundary conditions such as footwear, and (b) to use the CAI to investigate whether footwear influences gait asymmetry during running in a healthy, young cohort. Kinematic and kinetic data were collected for both legs of 15 subjects performing five barefoot and five shod over-ground running trials. Thirty continuous gait variables including ground reaction forces and variables of the hip, knee, and ankle joints were computed for each leg. For each individual, the differences between the variables for the right and left leg were calculated. Using this data, a principal component analysis was conducted to obtain the CAI. This study had two main outcomes. First, a sensitivity analysis suggested that the CAI had an improved sensitivity for detecting changes in gait asymmetry caused by external boundary conditions. The CAI may, therefore, have important clinical applications such as monitoring the progress of neuromuscular diseases (e.g. stroke or cerebral palsy). Second, the mean CAI for shod running (131.2 ± 48.5; mean ± standard deviation) was significantly lower (p = 0.041) than the CAI for barefoot running (155.7 ± 39.5). This finding suggests that in healthy, young adults gait asymmetry is reduced when running in shoes compared to running barefoot, which may be a result of improved neuromuscular control caused by changes in the afferent sensory feedback.

## Introduction

Falls are one of the main causes for fatal injury and hospitalization in older adults [1–3]. Identifying factors that contribute to falls has become an important objective in clinical geriatric research. The absence of footwear was identified as an important risk factor for the occurrence of falls in elderly adults [4]. The reduced risk of falls reported in the mentioned study concurs with other studies that assessed the effect of footwear on the likelihood of falls or balance [5–7]. In addition to mechanical factors potentially causing a reduced risk of falls when wearing footwear [8], it is also possible that footwear may alter the type or amount of afferent sensory feedback causing improved neuromuscular control. If footwear does in fact improve neuromuscular control, then such an influence might already be present in young, healthy adults, long before it may become clinically relevant in the prevention of falls. A feature that is often used to assess neuromuscular control of motion is the level of asymmetry between the contra-lateral limbs during gait. In fact, in many neurophysiological disorders such as stroke [9, 10], Parkinson’s disease [11], or cerebral palsy [12], gait asymmetry can be seen as one of the indicators of the severity of the condition.

One challenge when assessing gait asymmetry in healthy, young adults is that the kinematic and kinetic differences between the left and right lower limbs are rather small compared to the inherent movement variability. In addition, one could argue that gait asymmetry is a characteristic that applies to several body segments simultaneously [13–15], especially when investigating changes caused by external boundary conditions such as footwear. Therefore, a new asymmetry index, a *comprehensive asymmetry index* (CAI), is required that is especially sensitive to changes in gait asymmetry caused by external boundary conditions. Three actions can be taken in order to increase the sensitivity of the CAI: First, all available kinematic and kinetic data should be incorporated to provide an all-encompassing assessment of an individual’s lower limb gait asymmetry. This allows considering the moving human body as a whole system rather than analysing individual variables [16, 17]. Second, the waveforms of all gait variables should be normalized to their standard deviation waveform to account for asymmetry caused by the natural variability of the movement. This should be done since previous studies indicated that gait asymmetry may only be relevant when it exceeds the inherent variability of a gait variable [13, 18]. Third, a principal component analysis (PCA) can be used to filter out the covariate structure of gait asymmetry [16, 19]. This is based on the assumption that gait asymmetry observed in one variable can only occur if it is accompanied by asymmetries in other variables [19]. To give a simplified example: contra-lateral asymmetries in the knee joint angle can only occur within a given motion task, if ankle and/or hip angles change accordingly.

In summary, a CAI with enhanced sensitivity to detect gait asymmetry changes is required in order to investigate whether footwear influences the level of asymmetry between the contra-lateral limbs during gait. A reduction in gait asymmetry may support previous research indicating that footwear improves neuromuscular control. The new CAI should be tested on a highly automated movement, i.e. running, rather than more complex movements in which higher cognitive functions are more likely to interfere with the movement pattern and may potentially affect gait asymmetry.

Therefore, the objectives of the study were (a) to develop a *comprehensive asymmetry index* (CAI) that can be used to study changes in gait asymmetry caused by external boundary conditions such as footwear, and (b) to use the CAI to investigate whether footwear influences gait asymmetry during running in a healthy, young cohort. Based on the aforementioned studies, it was hypothesized that footwear decreases gait asymmetry as compared to barefoot running.

## Methods

### Study participants

Fifteen subjects were recruited for this study, seven females and eight males: age: 25.4 (SD 4.4) years; height: 1.74 (SD 0.07) m; mass: 71.2 (SD 8.4) kg. The subjects were healthy, with no neuromuscular or neurological disorders, and had no lower-extremity pain at the time of testing. All study participants provided written informed consent in accordance with the University of Calgary’s policy on research using human subjects. The study protocol was approved by the Conjoint Health Research Ethics Board of the University of Calgary.

### Data collection

Kinematic and kinetic data were collected while the subjects performed for each leg five barefoot and five shod heel-toe over-ground running trials (running speed: 4.00 ± 0.6 ms^−1^). A standard, neutral running shoe, without unique design features that potentially could have influenced gait asymmetry, was provided for each subject (New Balance 506; New Balance Athletic Shoe Inc., USA). A running trial was considered successful when the subject’s foot that was being tested landed within the edges of a force platform (Kistler Instrumente AG, Switzerland). The force platform was used to record ground reaction forces (GRFs) at a sampling rate of 2,400 Hz. At the same time, kinematic data were collected by means of a marker-based motion capture system having eight synchronized, digital, high-speed, infrared cameras (Motion Analysis Corporation, USA). Twenty-two retro-reflective markers were mounted on each study participant. Marker locations included the right and left anterior superior iliac spine, the right and left posterior superior iliac spine, and proximal, lateral, and distal aspects of the thigh and shank. To describe the foot motion, markers were placed at proximal and distal, and lateral locations of the test shoe and on corresponding locations on the bare foot. For the purpose of a neutral standing trial, additional markers were also placed on (and after the neutral trial removed from) the right and left greater trochanters, the medial and lateral knee joint, and the medial and lateral malleoli to define joint centres. A sampling rate of 240 Hz was used to record the trajectories of the markers.

### Data pre-processing

Cortex motion analysis software (Motion Analysis Corporation, USA) was used to reconstruct the trajectories of the 22 markers for each running trial. A fourth-order, low-pass, Butterworth filter was applied to the kinematic and kinetic data to filter out movement artefacts and measurement noise with cut-off frequencies of 6 Hz for kinematic data and 50 Hz for kinetic data [20]. Standard motion analysis software *(*KinTrak 7.0; Human Performance Laboratory, Calgary, Canada) was used to compute 30 time-continuous gait variables. The 30 variables included joint angles, joint moments, and joint angular velocities of the ankle, knee, and hip, as well as ground reaction forces in all three planes of motion: frontal, sagittal, and transverse (Table 1). Joint moments and GRFs were normalized to body weight. All variables were resampled to 101 time points representing 0 to 100% of the stance phase.

**Table 1:** Gait variables.

Segment	Variables (frontal, sagittal, and transverse planes)		
Hip joint	Angles [°]	Moments [BWm]	Angular velocities [°s^−1^]
Knee joint	Angles [°]	Moments [BWm]	Angular velocities [°s^−1^]
Ankle joint	Angles [°]	Moments [BWm]	Angular velocities [°s^−1^]
Centre of pressure	Ground reaction forces [BW]		

Time-continuous gait variables that were computed over the stance phase for each subject, leg, and shoe condition. These variable types were used for the comprehensive asymmetry index.

### Comprehensive asymmetry index

The following data-processing steps were conducted for each subject and shoe condition (i.e. barefoot and shod). First, the mean waveform for each of the 30 variables was calculated based on the five collected trials. Second, the mean waveform for each variable was divided by the average of the corresponding standard deviation waveforms. This was done to normalize the variables to account for asymmetry caused by the natural variability of the movement [13, 18]. Third, all normalized waveforms were vectorized into a 3,030-dimensional (30 variables x 101 time points) row vector, ***q***, by horizontally appending the waveforms. Hence, ***q***_***left_leg***_ and ***q***_***right_leg***_ incorporated all available information about an individual’s movement during the stance phase. Finally, a difference vector, ∆***q = q***_***right_leg***_ ***—q***_***left_leg***_, between the multi-dimensional row vectors of the right and left legs was calculated for each participant and shoe condition. The difference vector ∆***q*** quantified all measured aspects of asymmetry of the participants’ gait. Therefore, the vector norm of ∆***q*** (i.e. the Euclidean distance from the origin to ∆***q***) may serve as a single CAI of the study participants’ overall gait asymmetry.

However, ∆***q*** is a complex high-dimensional (3,030 dimensions) construct. It is possible that some components of ∆***q*** contain artefacts that appear to indicate asymmetry. These artefacts are actually the result of random fluctuations of the data due to the natural variability of the movement. The expected gait asymmetry changes within an individual were rather small and the signal-to-noise ratio is unfavourable. Relevant changes in the gait pattern and, therefore, in gait asymmetry between shoe conditions in one variable have to be interrelated with changes in the asymmetry of other variables [19]. It was speculated that the use of a PCA would allow increasing the sensitivity of the CAI to detect small changes in gait asymmetry. For the PCA, an input matrix ***M*** was created containing the difference vector for each individual with each shoe condition:

$$M=\;\left[\begin{array}{c} \Delta q_{1}\\ \vdots \\ \Delta q_{30} \end{array}\right]$$ (1)

The input matrix contained 3,030 columns (30 variables x 101 time points) and 30 rows (15 subjects x 2 shoe conditions). The PCA comprised the following steps: (1) calculation of the covariance matrix of ***M***; and (2) calculation of the eigenvectors and eigenvalues of the covariance matrix [21]. The eigenvectors represent the orthogonal principal component vectors (PC-vectors), ***p***. The PC-vectors are defined by the direction of the highest correlated variance in the data. Since in the current study the input matrix for the PCA contained the difference vectors (right-left) for each of the individuals, the variance in the matrix and the definition of the PC-vectors were due to the asymmetry of the individuals’ gait.

**Fig 1:**
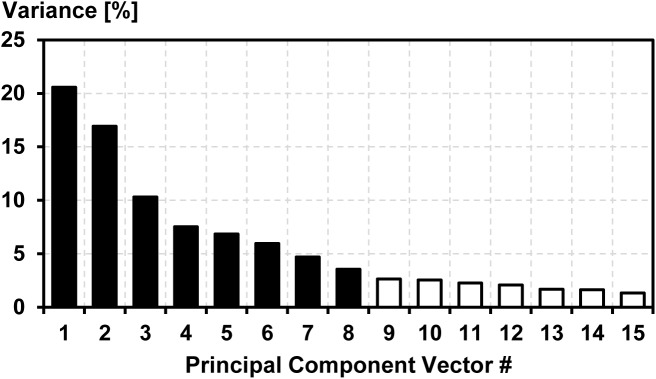
Eigenvalue spectrum. Eigenvalue spectrum of the first 15 principal component vectors that was used to determine the number of principal component vectors for the definition of the *comprehensive asymmetry index* (CAI). After the first eight eigenvalues (black bars) a drop can be seen. Hence, the first eight principal component vectors (k = 8) were used for the definition of the CAI.

The eigenvalue (EV) spectrum was assessed to determine a suitable number k of PC-vectors for the definition of the CAI. Within the first 15 EVs a drop is visible between EV8 and EV9 (Fig 1). Therefore, the first eight PC-vectors (k = 8) were expected to provide the best compromise between retaining as much correlated asymmetry as possible and filtering out uncorrelated noise [16].

The difference vectors ∆***q*** were then represented in a subspace spanned by the eight selected PC-vectors by projecting each difference vector ∆***q*** onto the PC-vectors:

$${\text{P}}_{\text{si}}=\Delta q_{s}\;\cdot \;p_{i}$$ (2)

where ***s*** indicates the study participants and ***i*** represents the number of the PC-vector. A subject- and condition-specific CAI was then calculated as the Euclidean distance from the origin using the projections (P_si_):

$${\text{CAI}}_{\text{s}}=\;\sqrt{\sum_{\text{i}=1}^{\text{k}}{\left({\text{P}}_{\text{si}}\right)}^{2}}$$ (3)

### Sensitivity analysis and statistics

To assess the sensitivity of the CAI, it was determined whether the difference vectors by themselves would be able to confirm the hypothesized difference in gait asymmetry between shod and barefoot running and how the CAI depended on the number k of PC-vectors used. Therefore, different variations of the CAI for each individual and shoe condition were calculated: (1) CAIs without PCA, using the vector norm (i.e. Euclidean distance) of the raw ∆***q*** only; (2) CAIs with PCA, based on all possible numbers of PC-vectors (k = 1…30). A paired samples t-test (*p*≤0.05; IBM SPSS Statistics 20, IBM Corporation, USA) was then used to assess the significance of the difference between the different mean CAIs for barefoot and shod running.

### Relevant asymmetry variables

The relevant asymmetry variables and their correlations were identified by analysing the loadings of the eight PC-vectors. The loading magnitude indicates the amount of variance in a variable that is captured by the corresponding PC-vector [22]. Since this variance was caused by gait asymmetry, variables with higher loadings contributed more to an asymmetrical gait. The loadings were multiplied with their corresponding EVs to weight the loadings according to the amount of variance/asymmetry covered by each PC-vector.

## Results

**Fig 2:**
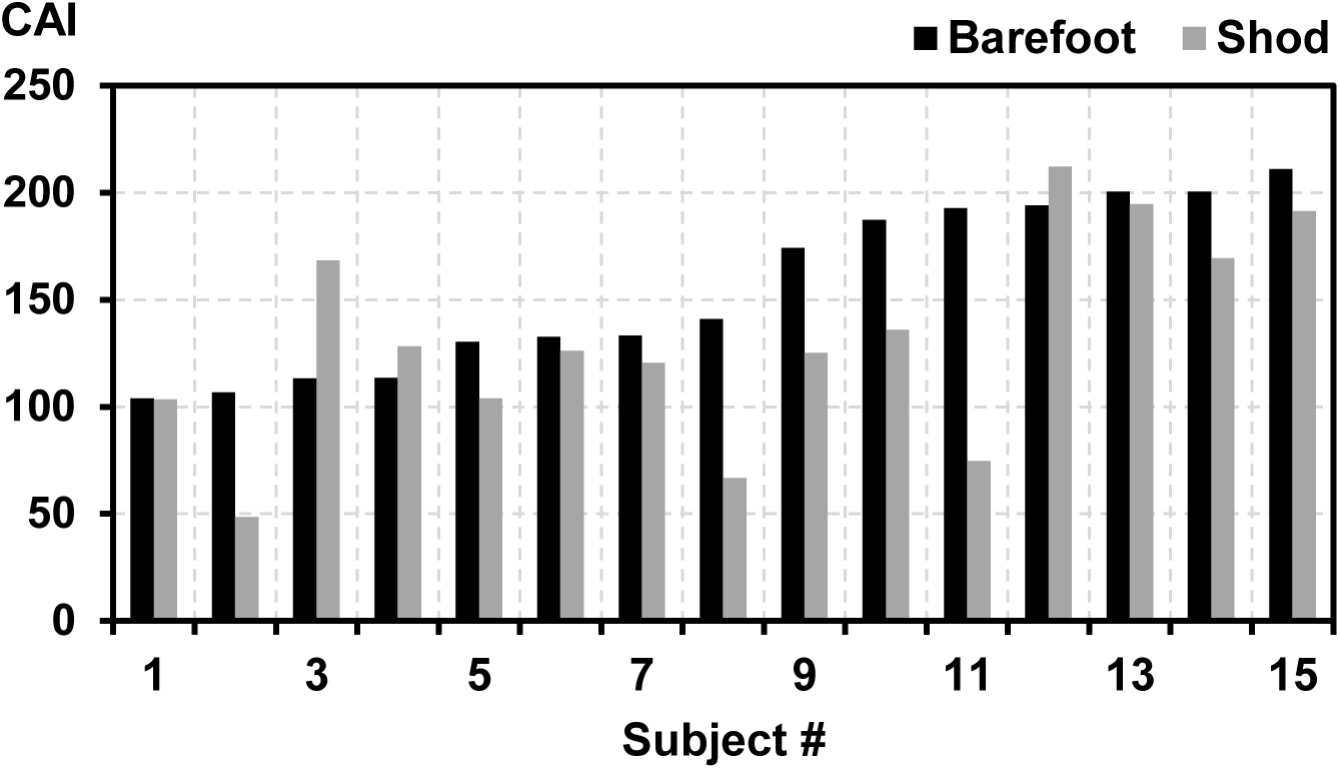
Subject-specific *comprehensive asymmetry index* (CAI) for barefoot and shod running. Study participants are arranged by increasing CAI for barefoot running. All CAIs calculated using eight principal component vectors (k = 8).

The eight PC-vectors that were used for the calculation of the CAI contained 76.4% of the overall asymmetry in all gait variables (Fig 1). The subject-specific CAIs for barefoot running ranged from 103.9 to 210.9, whereas the range for shod running was from 48.4 to 212.1 (Fig 2).

Averaged over all participants the CAI (k = 8) for running barefoot was 155.7 ± 39.5 (mean ± standard deviation) and for running in the shoe condition was 131.2 ± 48.5 (Table 2). The difference between the two conditions was significant (p = 0.041). Comparing barefoot and shod running using the CAI calculated as the direct Euclidean distance of the raw ∆***q*** to the origin (i.e. without filtering out uncorrelated asymmetries by the PCA) revealed no significant difference (p = 0.067; Table 2). The evaluation of how the CAI depended on the number k of PC-vectors used for the definition of the CAI showed that k ≤ 3 was not sufficient to detect significant asymmetry differences between barefoot and shod running (Table 2). For 4 ≤ k ≤ 8 and 12 ≤ k ≤ 13 the differences between the mean CAIs for barefoot and shod running were significant.

**Table 2:** Mean *comprehensive asymmetry indexes* (CAI) for barefoot and shod running.

k	Mean CAI Barefoot	Mean CAI Shod	p-Value
**∆q**	177.7 (SD 33.7)	157.9 (SD 39.1)	**0.067**
**1**	61.5 (SD 40.1)	68.6 (SD 48.2)	**0.302**
**2**	106.3 (SD 46.4)	84.0 (SD 43.5)	**0.084**
**3**	123.2 (SD 43.3)	98.6 (SD 41.2)	**0.060**
**4**	136.0 (SD 39.3)	104.0 (SD 45.4)	**0.020**
**5**	141.4 (SD 40.8)	113.9 (SD 48.6)	**0.045**
**6**	147.0 (SD 42.9)	121.4 (SD 48.4)	**0.042**
**7**	152.9 (SD 40.7)	126.3 (SD 47.6)	**0.031**
**8**	155.7 (SD 39.5)	131.2 (SD 48.5)	**0.041**
**9**	157.8 (SD 39.8)	135.1 (SD 46.8)	**0.061**
**10**	161.0 (SD 39.2)	136.9 (SD 47.0)	**0.052**
**11**	163.3 (SD 38.1)	139.6 (SD 46.2)	**0.059**
**12**	165.9 (SD 37.3)	142.0 (SD 43.5)	**0.042**
**13**	167.2 (SD 37.2)	144.2 (SD 42.9)	**0.050**
**14**	168.4 (SD 37.2)	146.2 (SD 42.8)	**0.064**
**15**	169.8 (SD 36.4)	147.4 (SD 42.8)	**0.060**
**16**	171.2 (SD 35.2)	148.7 (SD 42.4)	**0.058**
**17**	171.8 (SD 35.3)	150.1 (SD 42.6)	**0.069**
**18**	172.6 (SD 35.6)	151.0 (SD 42.8)	**0.072**
**19**	173.4 (SD 35.9)	151.8 (SD 42.3)	**0.073**
**20**	174.2 (SD 35.1)	152.8 (SD 42.1)	**0.072**
**21**	174.5 (SD 35.3)	153.7 (SD 42.0)	**0.078**
**22**	175.2 (SD 35.4)	154.3 (SD 41.6)	**0.075**
**23**	175.4 (SD 35.4)	155.3 (SD 41.1)	**0.083**
**24**	176.1 (SD 34.6)	155.7 (SD 40.9)	**0.071**
**25**	176.5 (SD 34.5)	156.2 (SD 40.8)	**0.071**
**26**	176.8 (SD 34.5)	156.7 (SD 40.4)	**0.072**
**27**	177.1 (SD 34.4)	157.1 (SD 40.0)	**0.072**
**28**	177.4 (SD 34.1)	157.4 (SD 39.7)	**0.069**
**29**	177.6 (SD 33.8)	157.6 (SD 39.5)	**0.067**
**30**	177.7 (SD 33.7)	157.9 (SD 39.1)	**0.067**

Mean *comprehensive asymmetry indexes* (CAI) and p-values (paired samples t-test) for comparisons between barefoot and shod running based on different CAIs calculated with the raw difference vector (∆q) and different numbers of principal component vectors (k = 1…30).

**Fig 3:**
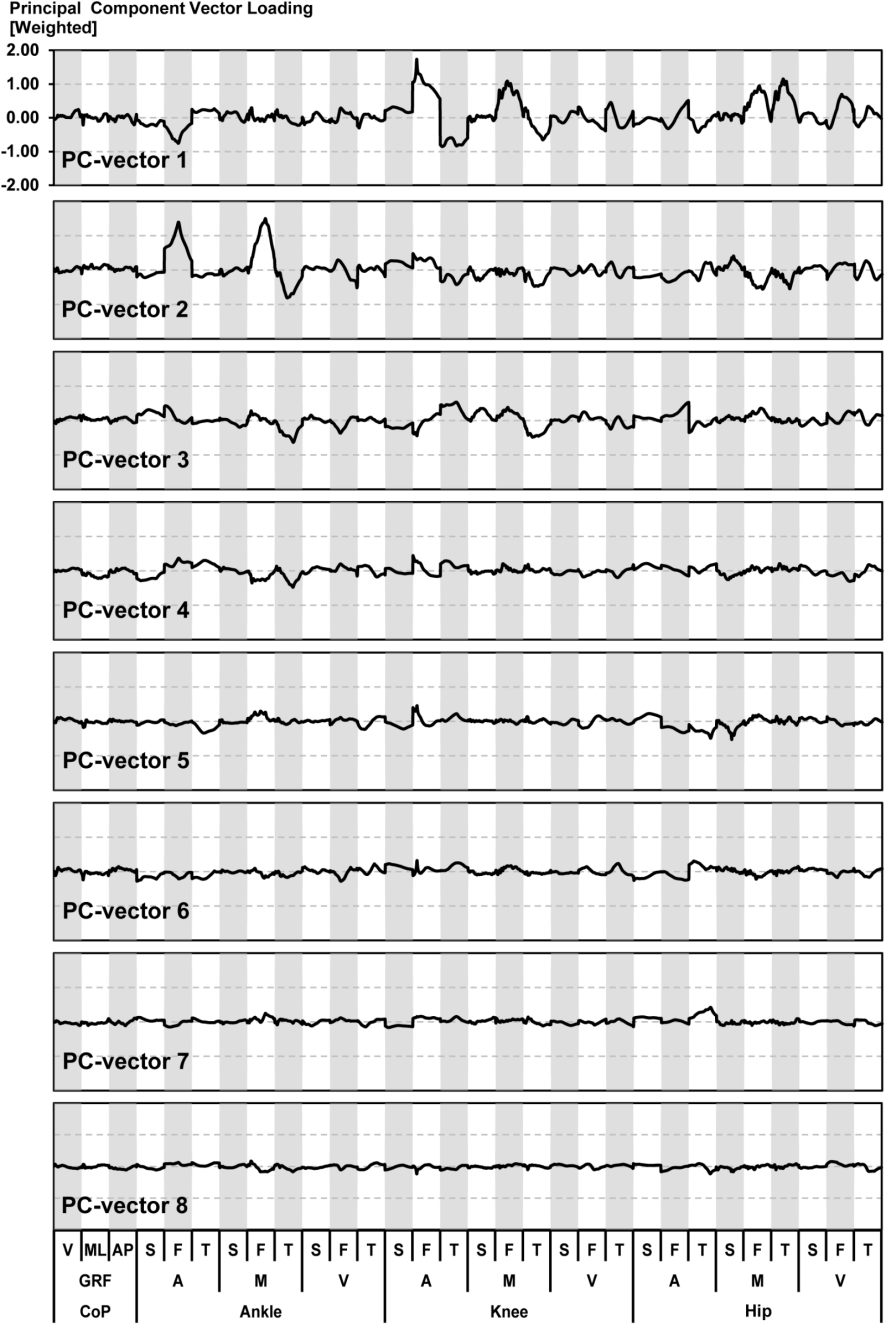
Weighted loadings of the first eight principal component vectors. These eight principal component vectors (PC-vectors) were used to calculate the *comprehensive asymmetry index* (CAI). Y-axes indicate the magnitude of the loading. X-axes represent the analysed biomechanical variables: V-Vertical; ML-Medial lateral; AP-Anterior posterior; GRF-Ground reaction force; CoP-Centre of pressure; S-Sagittal plane; F-Frontal plane; T-Transverse plane; A-Angle; M-Moment; V-Velocity.

The relevant asymmetry variables (i.e. variables with the highest PC-vector loadings) were mainly located in the ankle and knee joint (Fig 3). The frontal knee angle had the highest PC-vector loading (1.73) followed by the frontal ankle moment (1.50) and the frontal ankle angle (1.39). The PC-vector loadings showed correlations particularly between the frontal ankle angle/moment and the frontal knee angle/moment (PC-vector 1, PC-vector 2).

## Discussion

The current study had two main outcomes. First, a novel approach to quantify gait asymmetry was proposed that combined correlated asymmetries in multiple gait variables into one *comprehensive asymmetry index*, the CAI. The sensitivity analysis suggested that considering correlated asymmetries improves the sensitivity for detecting changes in gait asymmetry caused by external boundary conditions. This would be particularly useful when assessing the progression of clinical conditions such as cerebral palsy or the progress of rehabilitation treatments. The proposed method allowed to examining the structure of gait asymmetry by assessing the individual loadings of principal component vectors. Again, this has potential for clinical gait analysis and may contribute to a better understanding of the specific manifestations of a patient’s underlying condition, for example, in stroke and cerebral palsy patients. Second, the result of the CAI supported the hypothesis that even in healthy, young adults, gait asymmetry is reduced when running in shoes compared to running barefoot. This suggests that footwear seems to affect certain aspects of the neuromuscular control system that are involved in the coordination of the movements of left and right lower limbs.

### Comprehensive asymmetry index

The development of the CAI was motivated by the goal to provide a comprehensive asymmetry index with enhanced sensitivity for changes in gait asymmetry. Considering this main goal and the way it was implemented led to advantageous and disadvantageous characteristics of the proposed method, which will be discussed in the following paragraphs.

Since the CAI is a single value representing the totality of gait asymmetry of an individual (based on the measured variables), it facilitates direct comparisons between individuals with respect to overall gait asymmetry. The CAI offers no advantage, however, when it is necessary to quantify gait asymmetries of isolated variables (e.g. sagittal knee joint angle) at a specific time-point (e.g. at mid stance). In this case, other methods may provide a faster and more precise assessment of gait asymmetry [15, 23–26]. It is important to realize that CAIs can only be compared among individuals when they have been calculated using the same variables. Another limitation of the current method is that it is possible that unique gait asymmetries present in only one individual may not contribute sufficiently to be represented in the lower order PC-vectors. Therefore, if this method is applied as a diagnostic tool to quantify asymmetry in an individual patient, then both the PCA-filtered and direct Euclidean distance-based CAI should be assessed to ensure that the patient does not exhibit an unusual asymmetry pattern.

The results of the sensitivity analysis (Table 2) suggested that the PCA acted as a filter separating correlated from uncorrelated gait asymmetry variables [16]. Correlated asymmetries are more likely to contain actual differences in the movement pattern while uncorrelated asymmetries are more likely to contain a high proportion of noise [19]. Another advantage of determining the correlation structure of gait asymmetry using a PCA is that the resultant PC-vector loadings show the relevant asymmetry variables and their correlations. In fact, investigating the relevant asymmetry variables and their correlations suggested that the ankle and knee joint seemed to have the highest importance for the generation and compensation of gait asymmetry (Fig 3). Gait variables of the hip seemed to be less involved. Determining the relevant asymmetry variables and their correlation has potential for clinical gait analysis and may contribute to a better understanding of the specific manifestations of a patient’s underlying condition.

PCA has been used before when investigating gait asymmetry [14, 15, 24]. However, to the best knowledge of the authors, it has not yet been applied in the all-encompassing form that was set up in this study.

The CAI was based on data measured with a 3D motion capture system and a force platform during over-ground running. This experimental setup limits the amount of strides that can be measured and may also reduce the applicability of the CAI to monitor gait asymmetry in specific cases (i.e. a laboratory setting is required). Therefore, future studies should investigate the sensitivity of the CAI to detect gait asymmetry changes using data acquired with wearable sensors (e.g. accelerometers) to increase the amount of data that can be collected and the applicability of the CAI.

Because of the small sample size (15 study participants) and the recruitment of healthy individuals only, a systematic discussion of CAI values is not possible, and an actual non-pathological asymmetry range was not identified. Further studies should determine specific pathological and non-pathological ranges, as well as investigate how limb dominance, gender, or other external boundary conditions affect the CAI.

### Effect of footwear on gait asymmetry

Gait asymmetry in a healthy population has been documented in several studies [14, 15, 27]. Previous research has also reported an impact of footwear on the running kinematics and kinetics of healthy adults [28–30]. From a purely mechanical perspective, one would expect that wearing footwear, which may not be manufactured perfectly symmetrical, would either not affect or increase gait asymmetry. However, as pointed out in the introduction, previous studies indicated that footwear may improve neuromuscular control of motion. This might lead to a decrease in gait asymmetry as suggested by Vagenas and Hoshizaki [31] based on a limited set of isolated kinematic variables of the foot. The findings of the comprehensive analysis of this study support this hypothesis (Table 2).

Improved motor control mechanisms associated with wearing footwear might be a result of altered cutaneous sensory information of the plantar or dorsal surface of the feet [32–34]. Two recent review studies attested to the significance of plantar sensory feedback for the control of movement and supported the utilization of textured materials for improving perceptual-motor performance [35, 36].

The magnitude of the effect of footwear on gait asymmetry was subject-dependent (Fig 2). In fact, a few study participants (3 out of 15) even demonstrated an increase in gait asymmetry when running in shoes. De Wit et al. [28] reported a subject-depended impact of footwear on the kinematics and kinetics during running. However, it remains unknown which mechanisms cause these subject-dependent responses to footwear. One mechanism might be related to subject-specific sensitivity thresholds of the plantar or dorsal surface of the feet that may influence the afferent feedback to the neuromuscular control system [33].

## Conclusion

Footwear seems to reduce gait asymmetry during running in healthy, young individuals. Changes in the afferent sensory feedback to the neuromuscular control system may be a possible explanation for this observation.

## Supporting Information

S1 File Supplementary Data. Subject demographics, eigenvalue spectrum, subject-specific comprehensive asymmetry index (CAI) for barefoot and shod running calculated using the raw ∆***q*** and different numbers of principal component vectors (k = 1…30), and weighted loadings of the first eight principal component vectors.
(XLSX)

## Abbreviations

CAI: Comprehensive Asymmetry Index
EV: Eigenvalue
PCA: Principal Component Analysis
PC-vector: Principal Component Vector
